# Effects of a novel Nodal-targeting monoclonal antibody in melanoma

**DOI:** 10.18632/oncotarget.6049

**Published:** 2015-10-09

**Authors:** Luigi Strizzi, Annamaria Sandomenico, Naira V. Margaryan, Annalia Focà, Luca Sanguigno, Thomas M. Bodenstine, Grace S. Chandler, David W. Reed, Alina Gilgur, Elisabeth A. Seftor, Richard E.B. Seftor, Zhila Khalkhali-Ellis, Antonio Leonardi, Menotti Ruvo, Mary J.C. Hendrix

**Affiliations:** ^1^ Cancer Biology and Epigenomics Program, Stanley Manne Children's Research Institute, Ann and Robert H. Lurie Children's Hospital of Chicago, Chicago, IL, USA; ^2^ Department of Pathology, Northwestern University Feinberg School of Medicine, Chicago, IL, USA; ^3^ Department of Pediatrics, Northwestern University Feinberg School of Medicine, Chicago, IL, USA; ^4^ Robert H. Lurie Comprehensive Cancer Center, Northwestern University Feinberg School of Medicine, Chicago, IL, USA; ^5^ Istituto di Biostrutture e Bioimmagini del CNR and CIRPeB, Università Federico II di Napoli, Naples, Italy; ^6^ Dipartimento di Medicina Molecolare e Biotecnologie Mediche, Università Federico II di Napoli, Naples, Italy

**Keywords:** Nodal, cancer, antibody, ELISA, therapy

## Abstract

Nodal is highly expressed in various human malignancies, thus supporting the rationale for exploring Nodal as a therapeutic target. Here, we describe the effects of a novel monoclonal antibody (mAb), 3D1, raised against human Nodal. *In vitro* treatment of C8161 human melanoma cells with 3D1 mAb shows reductions in anchorage-independent growth and vasculogenic network formation. 3D1 treated cells also show decreases of Nodal and downstream signaling molecules, P-Smad2 and P-ERK and of P-H3 and CyclinB1, with an increase in p27. Similar effects were previously reported in human breast cancer cells where Nodal expression was generally down-regulated; following 3D1 mAb treatment, both Nodal and P-H3 levels are reduced. Noteworthy is the reduced growth of human melanoma xenografts in Nude mice treated with 3D1 mAb, where immunostaining of representative tumor sections show diminished P-Smad2 expression. Similar effects both *in vitro* and *in vivo* were observed in 3D1 treated A375SM melanoma cells harboring the active BRAF(V600E) mutation compared to treatments with IgG control or a BRAF inhibitor, dabrafenib. Finally, we describe a 3D1-based ELISA for the detection of Nodal in serum samples from cancer patients. These data suggest the potential of 3D1 mAb for selecting and targeting Nodal expressing cancers.

## INTRODUCTION

Melanoma is the most aggressive and deadly form of skin cancer with a median overall survival for advanced stage metastatic disease of less than 6 months [1]. Since the 1970's, dacarbazine (DTIC) has been the reference drug for patients with metastatic melanoma [2]. Despite the questionable survival benefit of DTIC therapy compared to supportive care [3], this drug is still listed as a therapeutic option for advanced stage or metastatic melanoma [4]. For decades no new therapeutic agent has been approved for metastatic melanoma by the FDA until a recent study, which showed survival benefit of a monoclonal antibody targeting a regulatory checkpoint, CTLA-4, in T-cells [5] and led to the approval of ipilimumab in 2011. A series of subsequent breakthroughs in targeted therapy also led to the approval of agents targeting BRAF (vemurafenib and dabrafenib) [6, 7] in patients harboring active BRAF V600 mutations in melanoma and of those targeting MEK (trametinib) [8], as well as the programmed death 1 pathway (PD-1) [9]. Enthusiasm, however, for the initial increased objective response rates in patients treated with these new targeting agents has diminished as follow-up data are demonstrating progression of disease and the inevitable development of resistance to these drugs. To address the challenge of drug resistance, combinatory approaches are under investigation and initial results are showing some improvement in progression free survival compared to monotherapy [10]; however, even with this approach, reactivation of MAPK, for example, can lead to early resistance [11]. Of special note are recent studies showing that targeting both PD-1 and CTLA-4 together in patients with metastatic melanoma resulted in higher rates of objective response and significantly longer progression-free survival than targeting CTLA-1 alone [12]. Continued follow-up will determine if this anti-immune checkpoint combinatorial approach will also lead to increased overall survival.

It appears, therefore, that the selective pressure exerted by the signaling pathway targeting agents can lead to activation or overexpression of alternative signaling events, all too common in melanoma, resulting in resistance and disease progression [13, 14]. Efforts to fine tune the clinical management of melanoma by determining ideal combinatorial regimens and timing of therapies will most likely lead to improvement in outcomes [15]. Nevertheless, the search for additional therapeutic targets and relative inhibitory agents will undoubtedly enrich the therapeutic armamentarium for melanoma by increasing the available options for concomitant or sequential targeting of pathways and growth factors as they become biologically relevant in melanomagenesis and disease progression [16].

Our studies have shown how Nodal, an embryonic growth factor of the transforming growth factor-beta (TGFB) superfamily, can play an important role in aggressive human cancer, specifically underlying tumor growth, metastasis and the cancer stem cell phenotype [17]. Typically, Nodal signaling occurs via binding to a receptor complex consisting of the EGF-like protein Cripto-1 and type I (ALK4/7) and type II (ActRIIB) activin-like kinase receptors [18]. This binding triggers intracellular phosphorylation of the Smad2/3/4 complex, which subsequently translocates to the nucleus, activating the transcription of genes that include Nodal itself and the Nodal antagonist, Lefty [18]. However, DNA methylation of the Lefty promoter in certain cancer cells including melanoma has been shown to represent a possible mechanism leading to unregulated Nodal expression and signaling [19].

Our findings as well as others have shown significant levels of Nodal expression in cancers of the prostate, breast and ovary, melanoma and others (Table 1). Since Nodal is not typically observed in most normal adult tissues, it has the potential as an attractive prognostic and predictive biomarker [20]. In fact, Nodal levels correlate with advanced stage disease in breast and prostate cancer and melanoma [21-23]. Studies have shown the feasibility of targeting Nodal *in vitro* and *in vivo* using either a polyclonal anti-Nodal antibody or shRNA approach, resulting in significant reduction in tumor cell activity and tumor volume [24, 25]. In a recent combinatorial study, we also describe the value of targeting Nodal in cells previously treated with DTIC [26]. Specifically, we showed that DTIC did not target the Nodal-positive subpopulation among the viable cells resistant to treatment. More importantly, we observed that tissue samples from patients with melanomas refractory to DTIC therapy showed positive immunostaining for Nodal, in both pre- and post-DTIC treated tumors. Also, *in vitro* experiments showed that combining DTIC treatment with a polyclonal anti-Nodal antibody decreased cell growth and increased apoptosis synergistically, at concentrations incapable of producing meaningful effects as monotherapy. Finally, we demonstrated that Nodal expression is maintained and targetable in BRAF(V600E) mutation-positive melanoma cells surviving anti-BRAF treatment with vemurafenib. Collectively, these observations strongly support ongoing efforts to develop clinically feasible approaches for targeting Nodal in melanoma as well as other aggressive cancers.

**Table 1:** 

Reference	Nodal detection
Adkins HB, et al. J Clin Invest, 2003 [47]	*In vitro* - human testicular, colon and breast cancer cells *In vivo* – human testicular cancer xenograft
Topczewska JM, et al. Nat Med, 2006 [29]	In vitro and *In vivo* – human melanoma
Postovit LM, et al. PNAS, 2008 [23]	In vitro – human melanoma and breast cancer cells *In vivo* – human breast cancer
Yu L, et al. Mod Pathol, 2010 [48]	*In vivo* – human melanoma
Lee CC, et al. Oncogene 2010 [49]	*In vitro* and *in vivo* – human gliomas
Lawrence MG, et al. Prostate, 2011 [22]	*In vitro* and *in vivo* – human prostate cancer
Strizzi L, et al. Breast Cancer Res, 2011 [21]	*In vitro* and *in vivo* – human breast cancer
Fu G and Peng C, Oncogene, 2011 [50]	*In vitro* – human ovarian cancer
Jamil S, et al. Int J Oncol, 2013 [51]	*In vivo* – human neuroblastoma xenograft
Duan W, et al. Oncotarget, 2015 [52]	*In vitro* – pancreatic cancer
Kong B, et al. Pancreatology, 2015 [53]	*In vivo* – human pancreatic cancer

Here, we describe the functional characterization of a novel mouse monoclonal antibody (mAb) specific to human Nodal, its biological effects on human tumor cells both *in vitro* and *in vivo,* and its potential as a capture antibody in an Enzyme Linked Immunosorbent based assay (ELISA) for the detection of Nodal in biological samples. This is the first description of a Nodal function-blocking mAb that could be further developed for clinical application.

## RESULTS

### Expression of Nodal in various human tissues

**Figure 1:**
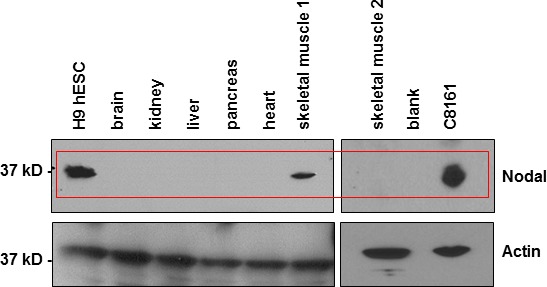
Nodal expression in normal human tissue lysates Commercially available Western blot grade normal human tissue lysates were analyzed for Nodal expression. Lysates from H9 hESCs were used as positive control for Nodal in the first lane. Nodal is not detected in lysates from normal human brain, kidney, liver, pancreas and heart. Low expression was detected in normal skeletal muscle sample 1, but no expression was detected in normal skeletal muscle sample 2. Nodal is highly expressed in C8161 human metastatic melanoma cells.

Our initial experiments tested a series of normal human tissue extracts for Nodal expression by WB analysis. Compared to Nodal detected in lysates from the H9 human embryonic stem cell line (H9) used as control, which is known to show robust expression of Nodal [23], we noted no appreciable Nodal protein expression in the major organs of brain, kidney, liver, pancreas or heart (Figure 1). A band with a similar molecular weight as that detected in H9 and C8161 cell lysates but with appreciably lower intensity was observed, however, in lysates from one of two skeletal muscle samples tested. Especially noteworthy are the findings from many laboratories reporting Nodal reexpression in several different types of human malignancies both *in vitro* and *in vivo* (Table 1). These data suggest that Nodal may represent a promising new therapeutic target specific to cancers.

### Generation and characterization of anti-Nodal mAbs

#### Production and selection of anti-Nodal mAbs

**Figure 2:**
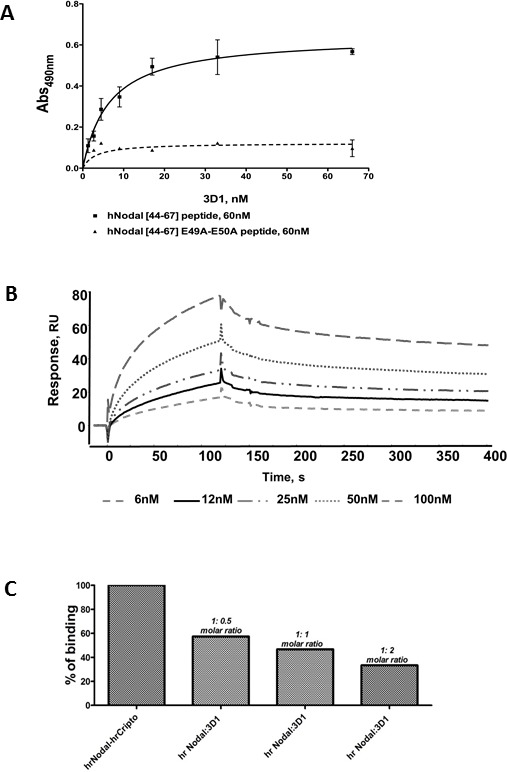
Characteristics of anti-Nodal 3D1 mAb **A.** ELISA-based binding assay of 3D1 mAb to coated *h*Nodal [44-67] and *h*Nodal [44-67] E49A-E50A. Peptides were coated at 0.18 μg/mL (60 nM). mAb 3D1 was tested at increasing concentrations between 1.0 and 67 nM. **B.** Overlay plot of SPR sensorgrams showing the interaction between 3D1 mAb and r*h*Nodal immobilized on a CM5 sensor chip. The interaction was monitored at concentrations of mAb ranging between 6.0 and 100 nM, obtaining dose-dependent binding curves. rhNodal was immobilized on a Biacore CM5 sensor chip and 3D1 mAb solutions at increasing concentrations were injected over the chip. **C.** Inhibition of the *rh*Nodal/*rh*Cripto-1 complex by SPR concentration-dependent competition assay. A plot of %binding *versus* increasing antibody concentrations is reported. *rh*Nodal was used at the fixed concentration of 5.0 nM whereas 3D1 was used at 1:0.5, 1;1 and 1:2 molar ratio.

3D1 production and generation has been previously described [27]. 3D1 binds the original antigen *h*Nodal [44-67], whereas it recognizes less robustly the mutated peptide variant *h*Nodal [44-67] E49A-E50A (Figure 2A), suggesting that it preferentially interacts with the two glutamic residues involved in the binding with Cripto-1 and potentially has a neutralization activity for the Nodal/Cripto-1 receptor complex interaction [28].

#### Binding of anti-Nodal mAb to rhNodal

The ability of 3D1 to bind *rh*Nodal was assessed by SPR by immobilizing the protein on Biacore sensor chips and injecting the purified antibody at increasing concentrations (Figure 2B). Association and dissociation rate constants together with thermodynamic dissociation constants (K_D_) were determined for each run and averaged. A K_D_ value of 1.42 nM was estimated in this way for the interaction between the 3D1 mAb and the protein.

#### Competition assays

Competition assays were performed using Biacore sensor chips derivatized with *rh*Cripto-1. For this test, we first assessed the binding between immobilized *rh*Cripto-1 and *rh*Nodal by injecting solutions of the Nodal protein at increasing concentrations [28]. We next incubated *rh*Nodal at 5 nM with 3D1 mAb at concentrations matching 1:0.5, 1:1 and 1:2 molar ratios (protein:mAb). As shown in Figure 2C, the 3D1 mAb at 5 nM (1:2 molar ratio) inhibited the binding of Nodal to Cripto-1 by approximately 70%. This is the first evidence demonstrating the ability of 3D1 to block the interaction between Nodal and Cripto-1 co-receptor.

### Function-blocking effects of 3D1 mAb *in vitro*

**Figure 3:**
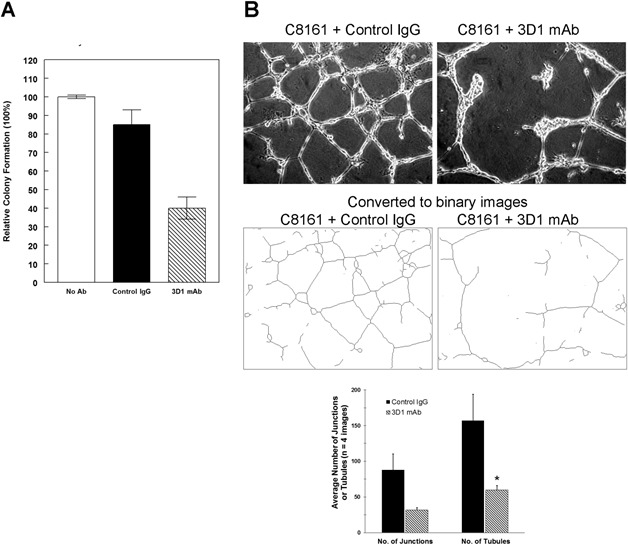
*In vitro* effects of anti-Nodal 3D1 mAb Results from anchorage independent growth assays **A.** show a significant reduction in anchorage independent growth of C8161 cells treated with 3D1 mAb compared to control cells. **B.** Results from vasculogenic network formation assay show a significant reduction in the ability to form junctions and tubules in C8161 cells treated with 3D1 mAb compared to control cells. (*P < 0.05.). Histograms represent mean values +/− SEM.

To determine whether the 3D1 mAb had the potential to affect C8161 melanoma tumor colony forming ability, untreated cells, or cells treated with either IgG control or 3D1 mAb for 72 hours were cultured in soft agar for three weeks (measuring anchorage independent growth). Cells treated with 3D1 mAb demonstrated a reduced ability to form non-adherent spheroidal clusters (signifying a decrease in anchorage independent growth) compared to untreated cells and cells treated with IgG control (Figure 3A). Nodal has been shown to underlie tumor cell plasticity associated with a cancer stem cell phenotype [29]. In particular, Nodal can induce phenotypic switching whereby melanoma cells are capable of assuming an endothelial-like phenotype via formation of capillary-like structures in a process known as vasculogenic mimicry (VM), which can be recapitulated in a three-dimensional (3D) culture system for *in vitro* studies [30]. When C8161 cells were treated with 4mg/ml of either 3D1 mAb or IgG control, and then grown in 3D cultures for 24 hours to measure their ability to engage in VM, the 3D1 mAb treated tumor cells were unable to form complete networks characteristic of VM, as measured by the reduced number of junctions and tubules using the AngioSys software package, compared to control (Figure 3B).

### 3D1 mAb effects on Nodal signaling and cell cycle regulators

#### Melanoma cells

**Figure 4:**
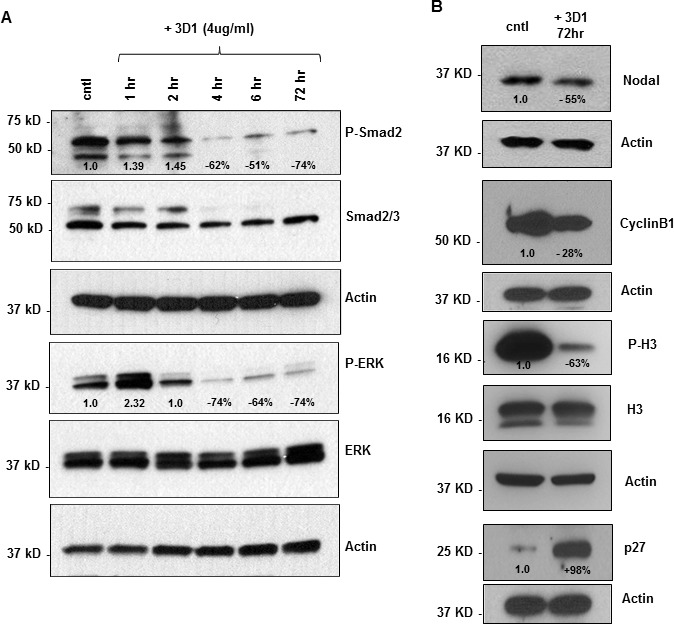
Effects of anti-Nodal 3D1 antibody on cell signaling and cell cycle related molecules **A.** Levels of P-Smad2 and P-ERK1/2 are reduced within 4hr of 3D1 mAb treatment (4 μg/ml) in C8161 human melanoma cells compared to IgG treated control. **B.** After 72 hr of 3D1 mAb treatment there is a reduction of Nodal, Cyclin B1 and P-H3 with a concomitant increase in p27 in C8161 cells compared to IgG treated control.

Treatment of C8161 cells with 4μg/ml of 3D1 mAb caused a reduction in phosphorylation of the Nodal related signaling molecules, Smad2 and ERK1/2, that was evident after 4 hrs and maintained through 72 hrs duration of the experiment (Figure 4A). Also, after 72 hrs of 3D1 mAb treatment, there was an associated reduction in Nodal expression (Figure 4B). Cyclin B1, a regulatory protein involved in mitosis and highly expressed in actively proliferating cells, was also reduced in C8161 cells treated with 3D1 mAb for 72 hrs (Figure 4B). Concomitantly, increases in p27, a cell cycle inhibitor protein that causes cell cycle arrest in G1 phase and the mitosis specific proliferation marker phospho-Histone H3 (P-H3) were observed in the same 3D1 treated C8161 cells *versus* control (Figure 4B).

#### Breast cancer cells

Based on our previous findings showing down-regulation of Nodal expression in human breast cancer cells results in decreased CyclinB1 and increased p27 [25], we extended our analysis to include the treatment of MDA-MB-231 human breast cancer cells with 3D1 mAb for the same time period. These results show a noteworthy reduction in Nodal expression as well as P-H3 by WB (Supplementary Figure 1).

Taken together, these results indicate that 3D1 mAb treatment diminishes Nodal expression, as well as downstream phosphorylation of Smad2 and ERK1/2, and reduces Cyclin B1 while increasing p27 in melanoma cells and that similar effects can be observed in breast cancer cells.

### Effect of 3D1 mAb *in vivo*

**Figure 5:**
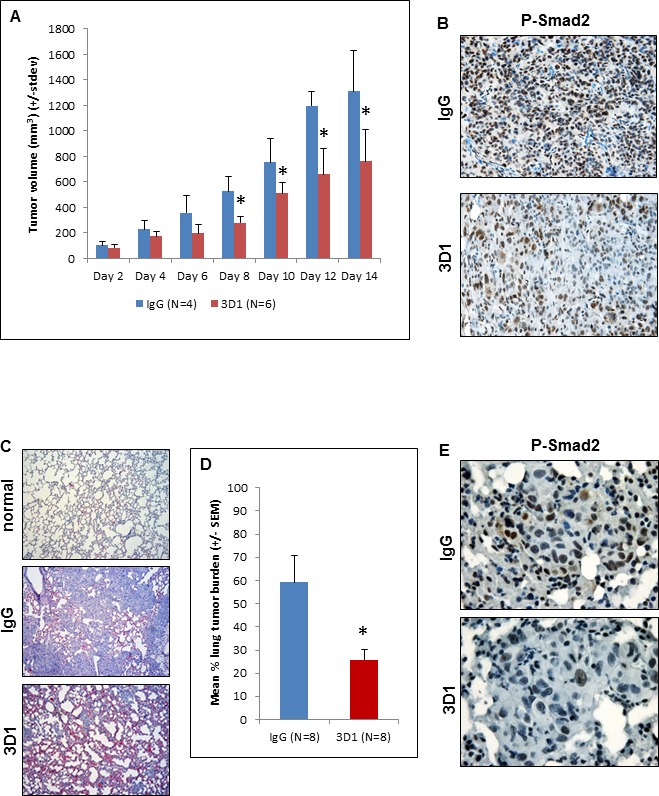
*In vivo* effects of anti-Nodal 3D1 mAb on C8161 human melanoma cells **A.** Significantly reduced tumor volumes are observed in C8161 Nude mice orthotopic xenografts treated with direct tumor injections of 3D1 mAb *versus* control IgG. Histograms represent mean values +/− SD. Representative IHC staining in **B.** shows reduced nuclear expression (activation) of Smad2 in C8161 orthotopic xenograft Nude mouse treated with 3D1 mAb compared to IgG control (20X original magnification). The potential for lung colonization (shown microscopically with H&E staining) of C8161 cells (after systemic introduction) in Nude mice **C.** (40X original magnification) is significantly reduced in animals treated with IP administration of 3D1 mAb vs IgG control **D.** Histograms represent mean values +/− SEM. Representative IHC staining in **E.** shows reduced nuclear expression (activation) of P-Smad2 in C8161 lung colony of a 3D1 mAb treated Nude mouse *versus* IgG control (63X original magnification). (*P < 0.05).

The inhibitory effects of 3D1 mAb described thus far *in vitro* led to the investigation of potential anti-tumor effects *in vivo*. To this end, several different xenograft models were established in Nude mice. First, an orthotopic model of cutaneous melanoma was established by injecting C8161 cells subcutaneously. Once appreciable tumors were detected in the mice, we initiated treatment with 3D1 mAb or control IgG via direct intratumoral injection. From days 8 to 14 of the experiment tumor volumes were significantly smaller in the 3D1 mAb treated mice compared to the IgG treated controls (Figure 5A). Sections of representative tumors formed by C8161 cells in these mice were analyzed to determine the effects of 3D1 mAb on Nodal signaling. IHC staining for P-Smad2 in these sections showed a significant reduction in the mean percentage (± SEM) of cells with strong positive nuclear P-Smad2 staining in the 3D1 mAb treated animals compared to the IgG treated control [IgG = 83% ± 8.2% (*N* = 4) *versus* 51.3% ± 3.3% (*N* = 4); *P* < 0.05] (Figure 5B). Since metastatic melanoma represents the most aggressive and clinically challenging form of the disease, we established a relevant metastatic model in Nude mice via systemic injection of C8161 cells. In this model, we have previously shown that C8161 cells readily colonize the lung within 7-10 days post-injection and that it is possible to target these cells using a commercially available polyclonal antibody against Nodal [24]. In the current study, when Nude mice treated with IgG control are compared to those treated with 3D1 mAb, a significant reduction in the mean percentage (± SEM) of lung tissue occupied by the C8161 colonies (lung tumor burden) was observed (Figure 5C) [3D1 mAb = 25.8% ± 4.6% (*N* = 8) *versus* IgG control = 59.3% ± 11.4% (*N* = 8); *P* < 0.05] (Figure 5D). Sections representative of C8161 lung colonies from 3D1 mAb and IgG treated mice were processed for IHC staining to determine the effects on Nodal related signaling. As with the subcutaneous tumors, the sections of lung from 3D1 mAb treated Nude mice showed significantly lower mean percentage (± SEM) of cells with nuclear localization of P-Smad2 compared to lung colonies in sections of the IgG control treated mice [3D1 mAb = 39 ± 8.9 (*N* = 6) *versus* IgG control = 59.4 ± 9.9 (*N* = 6); *P* < 0.01] (Figure 5E). Additional staining in these lung colony sections showed a significant decrease in the 3D1 mAb treated Nude mice of the percentage (+/−SEM) of cells staining for Cyclin B1 [3D1 mAb = 25.9% ± 9.4% (*N* = 6) *versus* IgG control = 38.8% ± 10.5% (*N* = 6); *P* < 0.001], along with a significant increase in 3D1 treated Nude mice of the mean percentage (± SEM) of cells staining for p27 [3D1 mAb = 39.5% ± 17% (*N* = 6) *versus* IgG control = 21% ± 15.3% (*N* = 6); *P* < 0.001] (Supplementary Figure 2).

**Figure 6:**
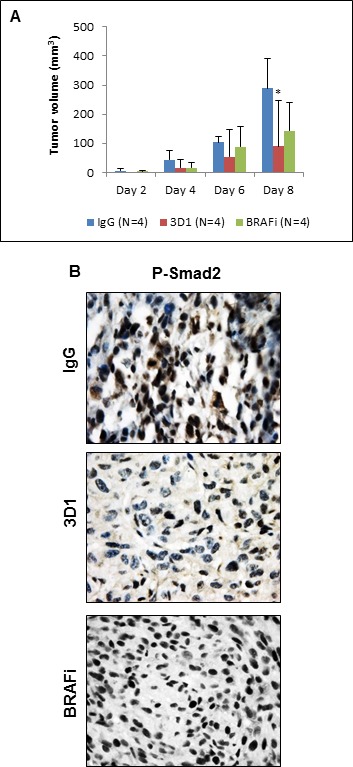
*In vivo* effects of anti-Nodal 3D1 mAb on A375SM human melanoma cells **A.** After 8 days, mean tumor volume of A375SM orthotopic Nude mice xenografts was significantly smaller in 3D1 mAb treated than in IgG control treated animals. Tumor volumes in dabrafenib (BRAFi) treated animals also showed a trend towards reduced tumor volumes compared to control. Histograms represent mean values +/− SD. **B.** Representative IHC of P-Smad2 showing nuclear staining in A375SM orthotopic xenografts in Nude mice treated with control IgG, 3D1 mAb or BRAFi (*P < 0.05).

Previously, we showed that Nodal expression in melanoma cells harboring the active BRAF mutation is unaffected when these cells are treated with a BRAF inhibitor (BRAFi) [26]. Here, we show that when the human melanoma cell line A375SM (containing the active BRAF mutation) is treated with the BRAFi dabrafenib, P-ERK1/2 is significantly reduced, as expected, but Nodal expression is relatively unaffected (Supplementary Figure 3A). However, Nodal expression is significantly reduced in A375SM cells when treated with 3D1 mAb (Supplementary Figure 3B). Furthermore, tumor volumes of A375SM orthotopic xenografts formed in Nude mice are significantly reduced in 3D1 mAb treated mice compared to BRAFi treated or IgG control treated mice (Figure 6A). IHC staining of P-Smad2 in representative tissue sections from these treated animals (Figure 6B) show a significant reduction of the mean percentage (± SEM) of nuclear P-Smad2 in 3D1 treated mice compared to mice treated with either the BRAFi or IgG control [3D1 = 28.1% ± 2.6% (*N* = 4); BRAFi = 68.7% ± 6.3% (*N* = 4); IgG = 50.2% ± 7.3 (*N* = 4); *P* < 0.05]. Collectively, these findings show the Nodal function-blocking activity and tumor diminishing effect(s) of 3D1 mAb treatment *in vivo*.

### ELISA detection of soluble Nodal

**Figure 7:**
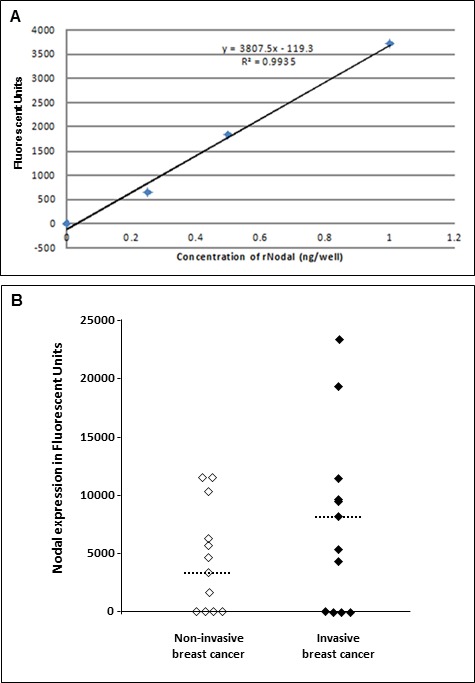
Sandwich ELISA assay developed to detect Nodal in the serum of breast cancer patients **A.** Illustrates a typical calibration curve using 3D1 mAb as the capture antibody for detecting recombinant Nodal; while **B.** depicts Nodal detected in patient's serum and with a trend for higher Nodal levels in the samples from patients with invasive compared to noninvasive breast cancer. (Dashed line = median level).

Since our previous study showed Nodal expression in tissue samples from a sizable cohort of breast cancer patients [21], the Nodal ELISA was developed to determine whether Nodal can be detected in serum samples from breast cancer patients. A typical calibration curve using a sandwich ELISA for detecting soluble recombinant Nodal is depicted in Figure 7A. Initially, conditioned medium from Nodal secreting H9 cells was employed to test the validity of the assay in evaluating secreted Nodal by ELISA. These findings supported a viable approach that led to the testing of biological fluids (i.e. serum) to identify secreted Nodal as a potential biomarker. Using this ELISA, Nodal was detected in 15/23 (65%) serum samples. When the serum samples were categorized based on the aggressiveness of the disease (Figure 7B), a trend for higher Nodal levels was noted in the invasive compared to noninvasive breast cancer patient sera [median for invasive breast cancer = 6807 (range: 0 - 23467; *N* = 12) *versus* median for noninvasive breast cancer = 3509 (range: 0 - 11541; *N* = 11)]. Thus, these results show that Nodal can be detected in sera from cancer patients with our novel 3D1 mAb based ELISA.

## DISCUSSION

Within just a few years the outcomes for the treatment of metastatic melanoma has evolved from little to no difference compared to palliative care, to substantially improved response rates and quality of life of affected patients - thanks to the introduction of novel targeted agents and immune checkpoint inhibitors [31]. Not all patients, however, are candidates for these promising new drugs. Moreover, development of drug resistance is often the culprit for failure of targeted therapy, which justifies ongoing efforts to shed light on the kinetics of *in vivo* resistance mechanisms. Molecular heterogeneity and high rates of somatic base mutations in melanoma can explain, at least in part, the selection of drug resistant clones of those melanoma cells that can ultimately lead to disease progression [32]. The cancer stem cell theory also suggests that within this continuously mutating heterogeneous tumor population reside subsets of cancer cells with stem cell-like characteristics that include drug resistance, and which have the potential for disease relapse and metastatic spread. In fact, growth factors and cell signaling events characteristic of stem cells have been shown to reemerge in some of these more stem cell-like cancer cells [33]. For instance, Nodal, a TGFB member, is predominantly expressed during early embryonic development and plays an important role in embryonic stem cell maintenance and during body axis establishment [18, 34]. Importantly, Nodal is not typically observed in most normal adult tissues (Figure 1). However, in this study a band corresponding to Nodal molecular weight was detected by WB in one of two skeletal muscle tissue lysates purchased from a commercial source. Since no information, other than the qualification of “normal tissue” was available from the vendor regarding clinical information or the context in which these samples were obtained, given the soluble nature of Nodal, one could speculate that Nodal, in this particular tissue sample, may have derived from an adjacent cancer tissue. Further investigation of Nodal expression in a third normal skeletal muscle tissue by IHC did not reveal any Nodal expression (Supplementary Figure 4). Nevertheless, Nodal is certainly reactivated in various types of advanced-stage cancers like metastatic melanoma and breast cancer, as well as others (Table 1). It is not known whether Nodal can play a role in conferring drug resistance in cancer cells, particularly with respect to the newly developed targeted agents, such as BRAFi. Indirect evidence, however, may support efforts towards investigating this possibility. For instance, EMT-like phenotype switching has been implicated in the development of drug resistance, including resistance to BRAF inhibitors [35], and Nodal has been shown to promote cellular invasion and EMT-like phenomena via phosphorylation of ERK1/2 [36]. In addition, the same TGFB receptor signaling shown to mediate resistance to the BRAFi vemurafenib [37] is employed for Nodal signal transduction in melanoma [38].

Studies have shown that it is possible to target Nodal in metastatic melanoma resulting in inhibition of cell aggressiveness *in vitro* and reduced tumor growth in *in vivo* xenograft models [23-25, 29]. More recently, we showed that the combination of a commonly used albeit poorly effective, chemotherapeutic agent DTIC with anti-Nodal antibodies results in synergistic decreased cell growth and increased apoptosis, at concentrations incapable of producing meaningful effects as monotherapy [26]. Moreover, that same study showed that Nodal expression was not affected in melanoma cells expressing a BRAF mutation when treated with a BRAFi. Thus, these observations suggest that a combinatorial approach involving Nodal targeting may improve treatment in a broader population of melanoma patients, regardless of BRAF mutation status.

In this study, we describe the production and functional validity of 3D1, a novel mAb capable of targeting and functionally blocking human Nodal. We show that 3D1 mAb is capable of robust binding of Nodal and that it binds in proximity to the Cripto-1 co-receptor interacting region encompassing Glu49 and Glu50 [39, 40]. The antibody also very efficiently blocks the *in vitro* binding of Nodal to Cripto-1*,* thus suggesting it can prevent the Cripto-1 co-receptor complex-dependent Nodal downstream signaling. Melanoma cells treated *in vitro* showed significant reduction of Nodal protein expression levels and reduction of the activated (phosphorylated) forms of SMAD2 and ERK1/2. This treatment was also accompanied by reductions in anchorage independent growth and vasculogenic mimicry, and decreased levels of the cell proliferation-associated molecules Cyclin B1 and P-H3, complemented by an increase of the cell cycle inhibitor p27. Most noteworthy, Nude mice xenograft models, both subcutaneous orthotopic and lung colonization models, treated with 3D1 mAb showed antitumor effects in terms of reduced tumor volume and lung tumor burden, respectively. Equally important, analysis of IHC staining for P-Smad2, p27 and Cyclin B1 in tumor sections confirmed the *in vitro* results. Also of significance is the effect of 3D1 treatment of breast cancer cells showing the down-regulation of Nodal expression and the accompanying decrease in P-H3. These findings validate key observations of a previous investigation where shRNA knockdown of Nodal expression in MDA-MB-231 and MDA-MB-468 aggressive breast cancer cells resulted in decreased CyclinB1 and upregulation of p27, concomitant with decreased tumorigenicity [25]. These data suggest that Nodal may represent a promising new therapeutic target specific to cancers.

From a translational perspective, in addition to types and rates of responses in patients for evaluating clinical outcomes, it is desirable that therapies have specific biomarkers for determining efficacy of treatment or disease relapse. For instance, obtaining circulating tumor cells from patients receiving targeting agents has been proposed for monitoring the emergence of new mutations, which in turn can be targeted with other available agents in a sequential or combinatorial manner [13]. Increased lymphocyte counts have also been suggested to serve as a potential biomarker for positive response(s) in patients treated with checkpoint inhibitors [41]. In this study, we describe the first application of 3D1 mAb in developing an ELISA based assay with the potential for detecting Nodal in biologic fluids. Since Nodal is a secreted protein, it is conceivable that patients with high Nodal expression during advanced stage tumor progression may also have detectable levels of circulating Nodal. In fact, here we show for the first time that this novel 3D1 mAb based Nodal sandwich ELISA is capable of detecting Nodal in serum from breast cancer patients. Future studies on a large cohort of serum samples from breast cancer patients at different stages of disease will be needed to determine whether serum Nodal expression can be used as a biomarker for staging in breast cancer or whether serum Nodal levels are affected by specific therapies. Nevertheless, we were able to determine from our preliminary analysis of 23 serum samples that Nodal tends to be expressed at higher levels in serum from patients with invasive breast cancer. With respect to melanoma, Nodal expression in melanoma tissue would not only justify selection of patients for anti-Nodal therapy, but this novel 3D1 mAb-based ELISA could provide the ability to assess pre-therapeutic Nodal levels, important for dosing and scheduling of the Nodal targeting agent for reasons mentioned previously. More importantly, these levels can provide a baseline for comparison with post-therapeutic levels of circulating Nodal useful as a surrogate biomarker indicative of a reduction of Nodal expressing melanoma cells and efficacy of anti-Nodal therapy.

Our previous studies have shown that Nodal has the potential as a prognostic and predictive biomarker in several types of human cancers, i.e. patients with high levels of Nodal expression have more aggressive disease, and targeting Nodal in aggressive cancer cells leads to reduced cell proliferation and tumor growth both *in vitro* and *in vivo* [20, 21]*.* Here, we describe a novel Nodal blocking mAb capable of anti-tumor effects demonstrated in melanoma models both *in vitro* and *in vivo*. Since Nodal is significantly expressed in melanoma and has been shown to play an important role in its aggressive behavior, this 3D1 mAb holds promise as a novel targeting agent. Nodal expression appears to be independent of the expression of currently targeted molecules and represents a potential alternative or complementary target in non-candidate and candidate melanoma patients for these new therapies. Future pre-clinical studies will determine the optimal dosing schedule with or without sequential or combinatorial associations with other targeting agents to define the best therapeutic approach in aggressive cancers expressing Nodal based on these new scientific findings.

## MATERIALS AND METHODS

### Reagents

TRIzol and restriction endonuclease enzymes were purchased from Invitrogen (Carlsbad, CA, USA). The protein G column was purchased from GE Healthcare. SPR analyses were performed on a Biacore 3000 instrument from GE Healthcare, using CM5 sensor chips and certified HBS buffer (20 mM HEPES, 0.15 M NaCl, pH 7.2, P20, 0.005%). Recombinant human Nodal (*rh*Nodal) and human Cripto-1 (*rh*Cripto-1) were from R&D Systems (Minneapolis, Minnesota, USA). The following antibodies were used for WB: rabbit anti-Nodal (H-110; Santa Cruz Biotechnology, Dallas TX); rabbit anti-P-Smad2 (44-244G; Life Technologies, Grand Island, NY); rabbit anti-Smad2/3 (07-408; Millipore, Lake Placid, NY); rabbit anti-P-p44/42 MAPK (P-ERK1/2) (9101S; Cell Signaling, Beverley, MA); rabbit anti-p44/24 MAPK (ERK1/2) (44-654-G; Life Technologies); mouse anti-actin (MAB1501; Millipore, Temecula, CA). The following antibodies were used for immunohistochemistry: goat anti-human Nodal antibody (LS-B3955; LifeSpan Biosciences, Seattle, WA) 1:150; rabbit anti-P-Smad2 (AB3849; Millipore) 1:100 - 1:250; anti-p27 (2552; Cell Signaling) 1:400; rabbit anti-CyclinB1 (ab32053; Abcam, Cambridge, MA) 1:200.

### Generation and analytical characterization of 3D1

The 3D1 mAb antibody generation and production have been previously described in detail [27]. Briefly, the 3D1 antibody was generated against region 44-67 of human Nodal, which has been reported as potentially involved in the Nodal-Cripto-1 interaction [39]. The antigen contains two glutamic residues, E49 and E50, crucially involved in the binding with the co-receptor Cripto-1. To select anti-Nodal antibodies able to recognize these two hot-spot residues, we screened clones using both *h*Nodal [44-67] and a mutated peptide, named *h*Nodal [44-67] E49A-E50A, in which E49 and E50 were replaced with two alanines. In typical ELISA assays, peptides were coated at 0.18 μg/mL (60 nM) and binding was probed using the antibody at increasing concentrations between 1.0 and 67 nM. Only antibodies binding the wild type peptide were further developed and tested for binding against the full length recombinant Nodal by both ELISA and Surface Plasmon Resonance (SPR). The 3D1 antibody was purified to homogeneity by two chromatographic steps including protein G affinity chromatography and gel filtration of bound fractions. The procedure for the binding between 3D1 and peptides has also been previously described in detail [27].

### Surface plasmon resonance (SPR) analyses

All SPR analyses were performed at 25°C using HBS as running buffer. Protein immobilization was carried out following the canonical amine coupling chemistry using the surface immobilization wizard procedure, operating at 5 μL/min. Channels were activated with EDC/NHS [N-(3-dimethylaminopropyl)-N'-ethylcarbodiimide hydrochloride(EDC)/N-hydroxysulfosuccinimide(NHS) [42] for 7 min; for the binding assays, *rh*Nodal, appropriately diluted in the pre-selected sodium acetate buffer pH 4.5, was coupled until a 4000 RU level was achieved. Residual reactive groups on the sensor chip surface were deactivated by addition of 1.0 M ethanolamine hydrochloride, pH 8.5. Antibody binding was tested at 20 μL/min injecting solutions of 3D1 (60 μL) in HBS at increasing concentrations (6.0 nM – 100 nM). A 10 mM NaOH solution was used to regenerate the chip surface. For competition assays, *rh*Cripto-1 was covalently immobilized at 5 μg/mL in 10 mM Sodium Acetate buffer pH 4.5 at a flow rate of 5 μL/min onto a CM5 sensor chip, reaching an immobilization level of 600 RU. *rh*Nodal at 5 nM in HBS was incubated with 3D1 at increasing concentrations, so to achieve Nodal:antibody final molar ratios of 1.0:0.5, 1:1 and 1.0:2.0. Each mixture was incubated for 30 min at room temperature before passing on the sensor chip. 60 μL of each solution was injected at a flow rate of 20 μL/min. On every biochip, an underivatized surface was prepared and used as control blank. All analyses were carried out at a flow rate of 20 μL/min, injecting a constant volume of 60 μL of antibody or competition solutions opportunely diluted in the HBS running buffer at various concentrations. The contact time was 3 minutes for the binding. Dissociations were monitored for at least 3 additional minutes. For every single analysis, experimental sensorgrams were aligned, subtracted of blank signals and overlapped. All mathematical manipulations and fitting were performed using the BiaEvaluation software, vers. 4.1 from GE Healthcare. All experimental data gave optimal fittings when processed assuming a 1:1 Langmuir binding interaction.

### Cell lines

The C8161 human melanoma cell line used was obtained from the University of Arizona, while the highly metastatic A375SM human melanoma cell line, which harbors the active BRAF(V600E) mutation [43], was a kind gift from Dr. Menashe Bar-Eli, University of Texas MD Anderson Cancer Center. The breast cancer cell line MDA-MB-231 was purchased from American Type Culture Collection (ATCC) (Manassas, VA). Cell lines were authenticated by short tandem repeat genotyping at the Lurie Children's Hospital of Chicago molecular diagnostics core, routinely tested for mycoplasma contamination with a PCR ELISA kit (Roche Applied Science) and maintained as previously described [44-46].

### Anchorage independent growth assay

C8161 melanoma cells (5,000 cells/well) were suspended in 0.35% agarose, RPMI 1640;10% serum with of either 4μg/ml mouse IgG control (Jackson ImmunoResearch, West Grove, PA) or 3D1 mAb (2.8 μg/ml) and were then overlayed onto a solidified layer of 0.5% agar; RPMI 1640; 10% serum in 6-well dishes. Cell clusters were allowed to form and were scored (50 cells or larger clusters) after 3 weeks in culture. Triplicate wells were averaged from separate experiments and presented as a percentage (mean ± SEM) of IgG control.

### Vasculogenic network formation assay

Three-dimensional matrices were prepared by spreading 75 μl of ice-cold Matrigel (average 12-15 mg/ml; Corning, Bedford, MA) into 12-well culture dishes and polymerized for one hour at 37°C. C8161 human melanoma cells (1 x10^5^ cells/well) were then plated onto the prepared Matrigel matrices in the presence of either 4μg/ml mouse IgG control or 3D1 mAb (2.8 μg/ml). No additional antibody was added. Tubular network formation was then observed after 24 hr and images captured digitally using a Zeiss model 25 inverted microscope (Carl Zeiss, Inc, Thornwood, NY) and Hitachi HV-C20 CCD camera (Hitachi Denshi Ltd., Woodbury, NY). Mean values were calculated from images of at least four different fields of both the IgG control and 3D1 mAb treated cultures, and then analyzed using the AngioSys software package (TCS CellWorks, Ltd., Buckingham, UK) with the mean number (± SEM) of junctions and tubules calculated from the analyzed fields.

### Western blot analysis

To determine the level of Nodal expression in brain, kidney, liver, pancreas, heart and skeletal muscle ready to use adult tissue extracts for Western blotting (WB) were purchased from Santa Cruz Biotechnology. For WB experiments of cell lines, whole cell lysates were prepared and quantified as previously described [29]. SDS-PAGE gel electrophoresis and WB were performed using standard techniques. PVDF membranes were blocked in 5% non-fat milk or 5% BSA and antibodies diluted in either 5% non-fat milk or 5% bovine serum albumin overnight at 4°C, depending on the manufacturer's recommendations. Signal was detected using West Pico chemiluminescence reagent (Thermo Fisher) and exposure to x-ray film.

### *In vivo* experiments

To evaluate the *in vivo* effects of 3D1 mAb, a metastatic melanoma lung metastasis model was established by injecting 250,000 C8161 cells intravenously in Nude mice. After 4 days during which cells were allowed to colonize the lungs, mice were separated into treatment and control groups. The treatment group received a total of 500μg of 3D1 mAb *versus* 500μg of irrelevant isotype IgG in the control group administered over 10 days (alternate day intraperitoneal injection (IP) of 100μg of either 3D1 mAb or IgG). At the end of the treatment period, mice were sacrificed and lungs harvested and processed for histologic evaluation of lung tumor burden and immunohistochemistry. Lung tumor burden was determined by evaluating the mean percentage of lung tissue occupied by C8161 in at least 4 separate fields at low power (10X objective) in lungs from at least 4 separate mice for each group and performed by 2 different observers. A final mean ± SEM was calculated from the separate means determined by the individual observers.

A second experiment was carried out to determine the *in vivo* effects of 3D1 mAb on primary melanoma. To this end, 500,000 C8161 cells were injected subcutaneously in Nude mice. Palpable tumors were then injected directly with either 700 μg of total 3D1 mAb (*N* = 6) or IgG control (*N* = 4) antibody (alternate day intratumoral injection of 100μg over 14 days). Mice were then sacrificed and subcutaneous tumors harvested and processed as previously described. To evaluate the effect of 3D1 mAb in the presence of the BRAF(V600E) mutation, a third orthotopic xenograft model was established in Nude mice via subcutaneous injection of approximately 150,000 A375SM human metastatic melanoma cells. Mice were treated IP with 3D1 mAb or IgG control, as described above, or 3mg/kg of dabrafenib.

### Immunohistochemistry

Four micron thick, formalin fixed, paraffin embedded tissue sections were prepared and immunohistochemistry was carried out on a Dako Plus autostainer (DAKO, Inc, Carpenteria, CA) as previously described [23]. Briefly, following antigen retrieval and blocking steps, sections were incubated in primary antibody for 60 mins, followed by appropriate biotinylated secondary antibody (Biocare Medical, Conrad, CA), and then streptavidin-horseradish peroxidase (Thermo Scientific Lab Vision). Color was developed with 3,3′-diaminobenzidine substrate (Thermo Scientific Lab Vision) and sections were counterstained with hematoxylin (Biocare Medical, LLC). As a negative control, adjacent serial sections were incubated with species appropriate irrelevant IgG (Jackson ImmunoResearch Labs) at the same concentration as primary antibodies. Similar to the lung tumor burden evaluation described above, mean percentage of positive staining ± SEM was determined by 2 different observers, each calculating the ratio of positive cells/total number of cells × 100 for 4 separate high power fields (63X objective) from at least 2 different mice per group.

### Sandwich ELISA

By epitope mapping of commercially available Nodal antibodies and 3D1 mAb, the latter was chosen as the capture antibody for coating ELISA dishes, while a rabbit monoclonal anti-Nodal (Abcam) served for detecting the bound Nodal. The immune complex was subsequently quantified using a horseradish peroxidase-conjugated anti-rabbit antibody in conjunction with QuantaRed enhanced chemifluorescent horseradish peroxidase substrate (ThermoFisher). The established sandwich ELISA assay could detect Nodal as a recombinant protein in conditioned media from human embryonic stem cells and in biological fluids with a detection limit (calculated using recombinant Nodal) of 75 pg/well. Human serum samples from breast cancer patients were purchased from BioOptions (Brea, CA).

## SUPPLEMENTARY MATERIAL FIGURES


