# Supplementary material for: Effects of a novel Nodal-targeting monoclonal antibody in melanoma
Source: Oncotarget. 2015 Oct 9;6(33):34071–86. doi: 10.18632/oncotarget.6049 (PMC4741437; doi:10.18632/oncotarget.6049)
Supplement: Supplementary file 1 [file oncotarget-06-34071-s001.pdf]

Effects of a novel Nodal-targeting monoclonal antibody in melanoma

Supplementary Material

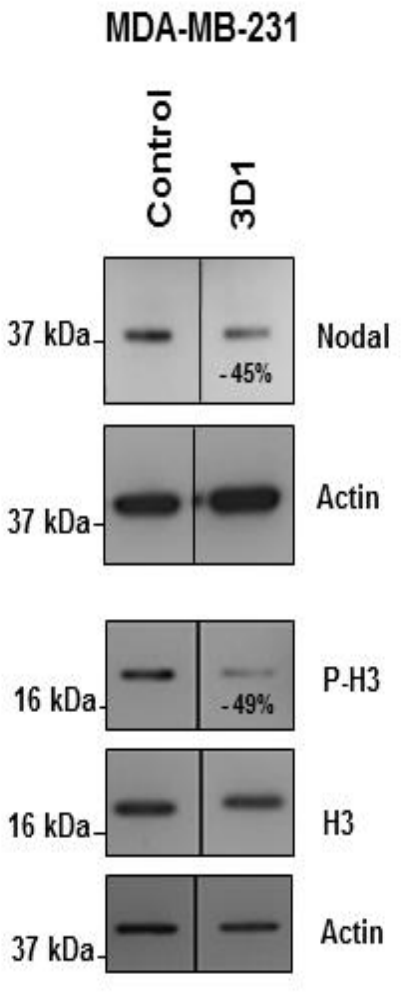

Supplementary Figure 1: Effects of anti-Nodal 3D1 antibody on MDA-MB-231 breast cancer cells. Results from Western blot analysis show a 45% reduction in Nodal and a 49% reduction in the level of the proliferation/mitosis marker P-H3 in MDA-MB-231 breast cancer cells treated for 72 hrs (4μg/ml) of 3D1 compared to the IgG control treated cells.

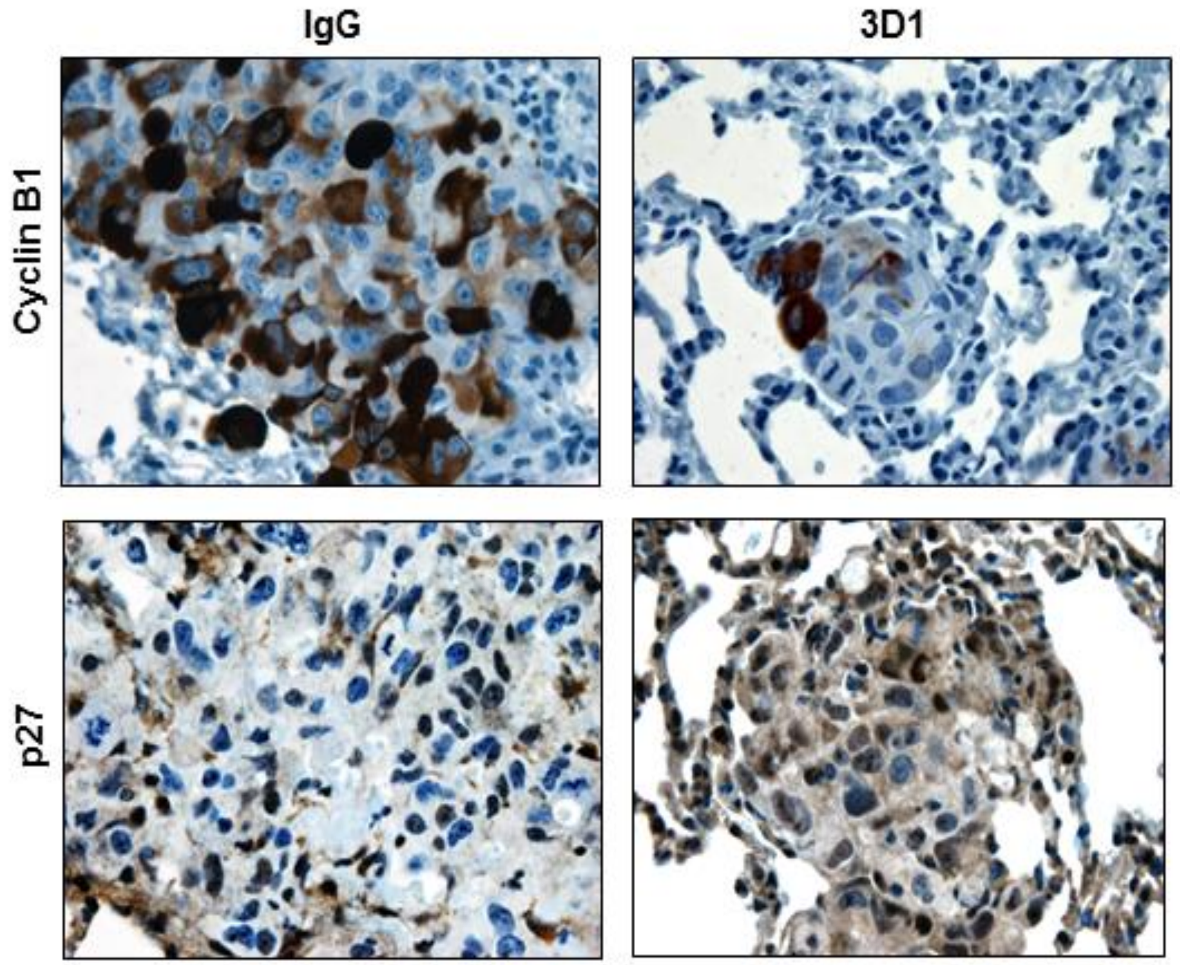

Supplementary Figure 2: Representative IHC results showing expression (brown stain) of Cyclin B1 and p27 in C8161 lung colony in Nude mouse +/- 3D1. Cyclin B1 expression is decreased in 3D1 treated tumors compared to IgG control. In contrast, p27 expression is increased in the 3D1 treated Nude mouse compared to control. (Original magnification 63X objective).

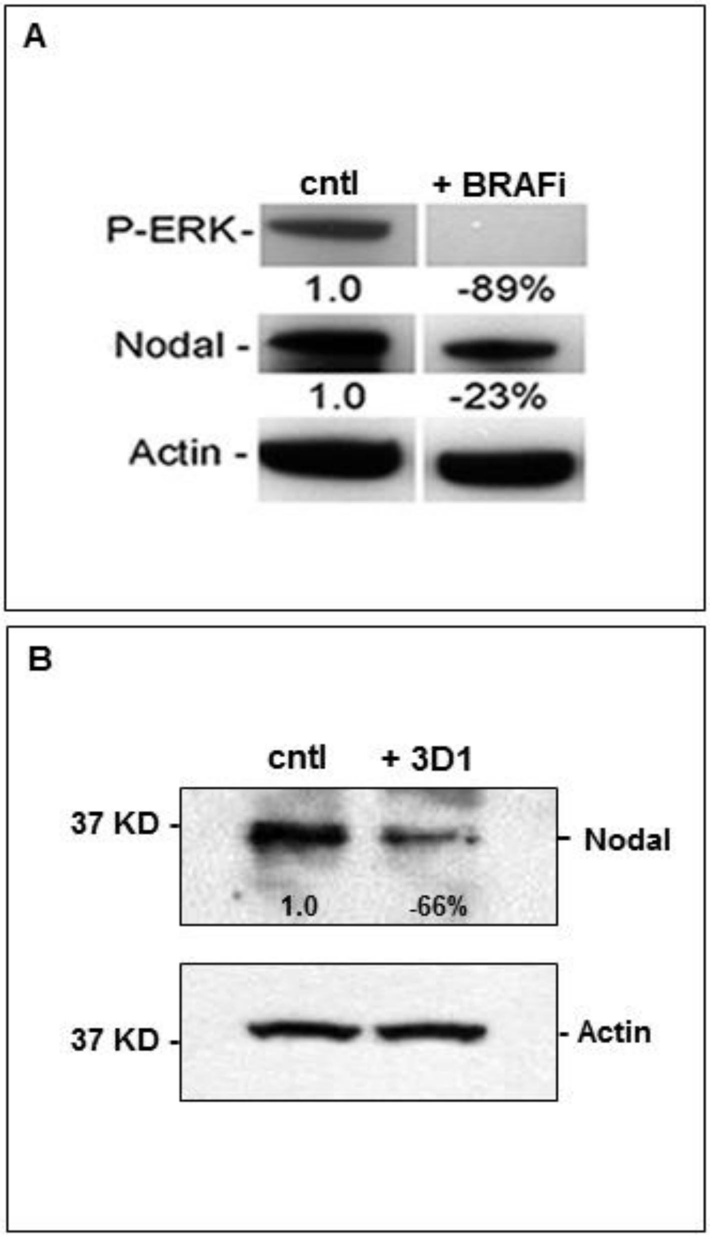

Supplementary Figure 3: Effects of BRAFi (dabrafenib) or 3D1 in A375SM cells. A) After 72 hr, ERK1/2 activity (P-ERK1/2) is significantly reduced in A375SM human melanoma cell line, which harbors the active BRAF mutation when treated with 10nM of the BRAFi, dabrafenib compared to control, while Nodal is only minimally affected. In contrast, 72h 3D1 treatment of A375SM **B**) showed a more dramatic reduction in Nodal expression compared to control.

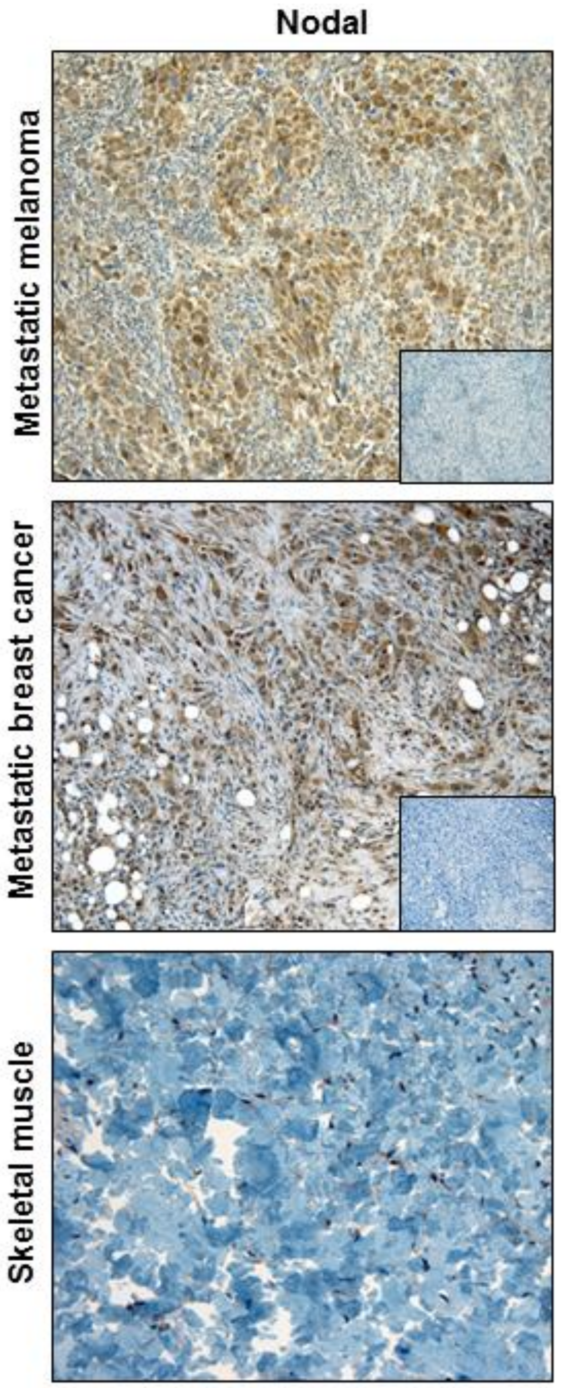

Supplementary Figure 4: Additional results for Nodal expression in normal skeletal muscle: Immunohistochemistry analysis shows Nodal staining in metastatic melanoma and metastatic breast cancer tissue sections (insets = negative control with irrelevant isotype IgG). In contrast, no appreciable staining for Nodal was detected in a skeletal muscle tissue section.
